# Influences of pH and EDTA Additive on the Structure of Ni Films Electrodeposited by Using Bubble Templates as Electrocatalysts for Hydrogen Evolution Reaction

**DOI:** 10.3390/membranes11030165

**Published:** 2021-02-27

**Authors:** Xiangtao Yu, Jun Yang, Xiangyu Ren, Zhuyin Sui

**Affiliations:** 1 Collaborative Innovation Center of Steel Technology, University of Science and Technology Beijing, Beijing 100083, China; yangjunbeike@163.com (J.Y.); G20199290@xs.ustb.edu.cn (X.R.); 2 School of Chemistry & Chemical Engineering, Yantai University, Yantai 264005, China; suizy@ytu.edu.cn

**Keywords:** hydrogen bubble template, electrocrytallization, porous Ni films, structure evolution, hydrogen evolution reaction

## Abstract

The structure of Ni films is essential to their electrocatalytic performance for hydrogen evolution reaction (HER). The pH value and EDTA (ethylene diamine tetraacetic acid) additive are important factors for the structure control of electrodeposited metal films due to their adjustment of metal electrocrystallization and hydrogen evolution side reactions. The structures of Ni films from 3D (three-dimensional) porous to compact and flat structure are electrodeposited by adjusting solution pH values or adding EDTA. It is found that when pH value increases from 7.7 to 8.1, 3D porous films change to compact films with many protrusions. Further increasing the pH value or adding 0.1 M EDTA causes compact and flat films without protrusions to appear. When pH ≤ 7.7, hydrogen bubbles with large break-off diameter are easily adsorbed on film surface acting as porous structure templates, and the electroactive ion species, Ni^2+^ and Ni(NH_3_)*_n_*^2+^ complexes with low coordination number (*n* ≤ 3), possess high reduction overpotential, which is beneficial to forming protrusions and smaller particles. So, porous Ni films are electrodeposited. In solutions with pH ≥ 8.1 or 0.1 M EDTA, Ni(NH_3_)*_n_*^2+^ complexes with high coordination number (6 ≥ *n* ≥ 3) and hexadentate chelate are formed. Due to the improved wettability, bubbles with a small break-off diameter rapidly detach the film surface resulting in strong stirring. The reduction overpotential is reduced, leading to the formation of larger particles. Therefore, the solution leveling ability increases, and it is difficult to form protrusions, thus it forms a compact and flat film. The 3D porous film exhibits excellent catalytic performance for HER due to the large catalytic activity area.

## 1. Introduction

**Figure 1:**
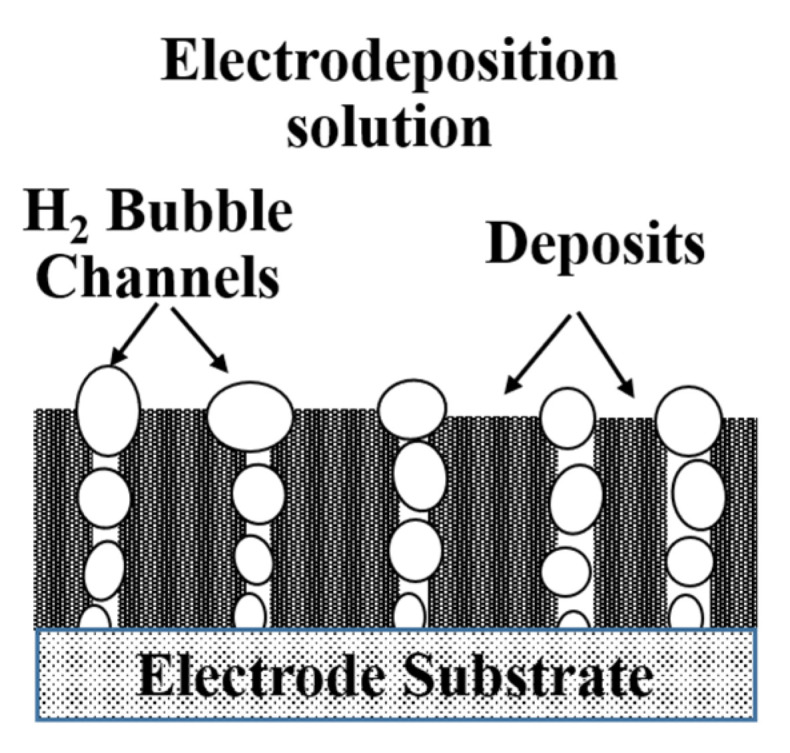
The schematic illustration of hydrogen bubble dynamic template.

Binder-free self-supporting porous metal film possessing a large active surface area and good wettability could accelerate the electron transfer, ion transportation, and bubble detachment, which results in excellent electrochemical activity and stability in electrocatalysis, fuel cells, batteries and other electrochemical energy conversion fields [1,2,3]. Among many self-supporting porous film preparation methods, the electrodeposition method is considered to be one of the most promising methods because it is convenient and easy to control [4,5]. Especially, the hydrogen bubble dynamic template (HBDT) method, which does not require subsequent removal of the template, has received more and more research attention in recent years [1,6,7]. It is based on the hydrogen bubble channels generated by the strong hydrogen evolution side reaction at a high current density during the metal electrodeposition process as a dynamic template, and deposits grow along the bubble channel template. When the bubbles break away, a self-supporting porous film is formed (Figure 1) [8]. Various metal and alloy porous films with good electrochemical properties for electrochemical energy conversion, such as Cu [9,10], FeP [11], CoP [12,13], NiCoP [14], were prepared by using the HBDT method. However, it is difficult to control the porous structure of the film electrodeposited by HBDT method, and the mechanism is unclear.

Metal electrocrystallization and hydrogen evolution side reaction are the two main reactions during the process of electrodepositing porous materials by using the HBDT method. The characteristics of electroactive ion species for metal electrodeposition, such as molecular structure, exchange current density, and reduction overpotential, could affect the nucleation and growth of metal crystal, which in turn determines the microstructure [15,16]. Kim et al. [17] found that PEG (polyethylene glycol) and MPSA (3-mercapto-1-propane sulfonic acid sodium salt) could form complexes with Cu^2+^ to change the size, shape, and density of Cu deposits, which is beneficial to forming 3D interconnected porous Cu films. Nam et al. [18] considered that BTA (benzotriazole, C_6_H_4_N_3_H) and NH_4_^+^ could also be complexed with Cu^2+^ to change the electrocrytallization behavior. Consequently, porous Cu films with needle-like nanodendrites or grape-like particles were obtained. On the other hand, behaviors of bubbles as templates on the film surface, such as bubble adsorption, coalescence, growth, and detachment, could affect the porous structure [2,19]. Additionally, the stirring effect generated by the bubble detachment could change the electrocrystallization kinetic conditions to affect the film structure [7,20].

pH value is an important factor to adjust the form of complexes, especially in solutions containing NH_4_^+^. M(NH_3_)*_n_*^2+^ complexes with different coordination number (n) are formed at different pH values [16]. Moreover, the H^+^ concentration in the solution that has a great impact on the hydrogen evolution side reaction is determined by the pH value. However, the dependence of pore structures of Ni films, which are a promising electrocatalyst for HER, on the pH value was not studied. On the other hand, EDTA could complex with metal ions to form a stable six-coordinate chelate, thereby changing the metal electrocrystallization behavior [21]. Therefore, it is significant to study the influences of pH value and EDTA additive on the porous structure of Ni films electrodeposited by using the HBDT method. 

In this work, the influences of pH value and EDTA additive on the surface morphology and crystal structure of electrodeposited Ni films were studied. The structure evolution mechanism was analyzed based on the two aspects of hydrogen bubble evolution and metal electrocrystallization behaviors. Finally, the catalytic performance for HER of the electrodeposited Ni films with different structure was also evaluated. 

## 2. Experimental

### 2.1. Electrodeposited Ni Films by Using the HBDT Method

In order to obtain a larger current density of −1 A cm^−2^, a CHI 760C electrochemical workstation connected to a CHI 660 current amplifier (CH Instrument, Inc., Shanghai, China) was used to electrodeposit Ni films in a standard three-electrode cell. The working electrode and counter electrode were a 10 mm × 10 mm Cu foil and a 25 mm × 25 mm Pt foil, respectively. The reference electrode was a saturated calomel electrode (SCE). Before electrodeposition, the Cu foil is polished with 2000 mesh sandpaper, and then washed with dilute hydrochloric acid and deionized water. The basic solution for Ni electrodeposition consisted of 0.2 M NiCl_2_, 2 M NH_4_Cl, and 1 M NaCl. pH was adjusted to 3–9.1 by dilute HCl and NaOH solutions. In order to fully complex the Ni^2+^, a large amount of EDTA was introduced to the basic solution, and the pH value was adjusted to 3 [21,22]. The electrodeposition was performed in basic solutions with different pH values or adding 0.1 M EDTA for 30 s at 30 °C. Fresh double-distilled water was used in all experiments, and all reagents were of analytical grade.

### 2.2. Characterization of Electrodeposited Ni Films

The microstructure and crystal structure of electrodeposited Ni films were observed by Field Emission Scanning Electron Microscopy (FESEM) (JEOL JSM-7001F, Akishima, Japan) and X-ray diffraction (XRD) (RIGAKU D/max-RB, Osaka, Japan), respectively. The electrodeposited Ni films were dissolved by concentrated nitric acid, and then diluted to 100 mL by double-distilled water. Electrodeposited Ni content was calculated based on Ni concentration measured by inductively coupled plasma atomic emission spectrometer (ICP-AES) (PE Optima 6300DV, Waltham, MA, USA). Finally, the current efficiency of hydrogen evolution (*η*_H2_) was calculated by Equation (1) [16,19]:

(1) $$\eta_{H2}=1-\frac{2CF}{ItM_{Ni}}\times 100\%$$

where *C* is the amount of electrodeposited Ni in mass (g), *F* is the Faraday constant (96485 C mol^−1^), *I* is current intensity (A), *t* is electrodeposition time (s), and *M*_Ni_ is the molecular mass of Ni (58.7 g mol^−1^). NH_4_^+^ concentration in basic solutions with different pH values were measured by ion chromatography. In order to evaluate the wettability of the solutions on the substrate, the contact angles and surface tension were measured by contact angle tester (Data physics Co. Ltd., GER OCA-20, Feldstadt, Germany). *E*-pH diagram of 0.2 M NiCl_2_, 2 M NH_4_Cl, and 1 M NaCl solution system at 25 °C was calculated by the common HSC chemistry software to analyze the existence forms of Ni^2+^ in different pH value regions [23,24]. The special surface area of Ni films has been quantitatively measured by the BET (Brunauer–Emmett–Teller) method. The surface chemical compositions and states of the Ni film were measured by X-ray photoelectron spectroscopy (XPS) using an ESCALAB 250Xi X-ray photoelectron spectrometer.

### 2.3. Electrochemical Analysis

The polarization curve measurements for Ni electrodeposition were performed in basic solutions with different pH values or different NaCl concentrations. The potential range was −0.9 V to −1.4 V and the scan rate was 10 mV s^−1^. To evaluate the catalytic activity and electrochemical activity area of various Ni films for HER, polarization curve, electrochemical impedance spectroscopy (EIS), and cyclic voltammetry (CV) measurements were carried out in 1 M NaOH solution. The potential range of the polarization curve measurement for HER was from −0.7 to −1.3 V, and the scan rate was 10 mV s^−1^. The EIS measurements were performed in frequencies ranging from 100 kHz to 0.01 Hz at an overpotential of 250 mV. The potential range of the CV measurement was from −0.31 to −0.46 V, and the scan rate was 10 mV s^−1^. Chronopotentiometry (CP) curves of Ni electrodeposition were recorded in different solutions at −1 A cm^−2^. The long-term electrolysis was performed in 1 M NaOH at 100 mA cm^−2^ for 1400 min. All experiments were repeated at least twice under the same condition to ensure reproducibility and accuracy.

## 3. Results and Discussion

**Figure 2:**
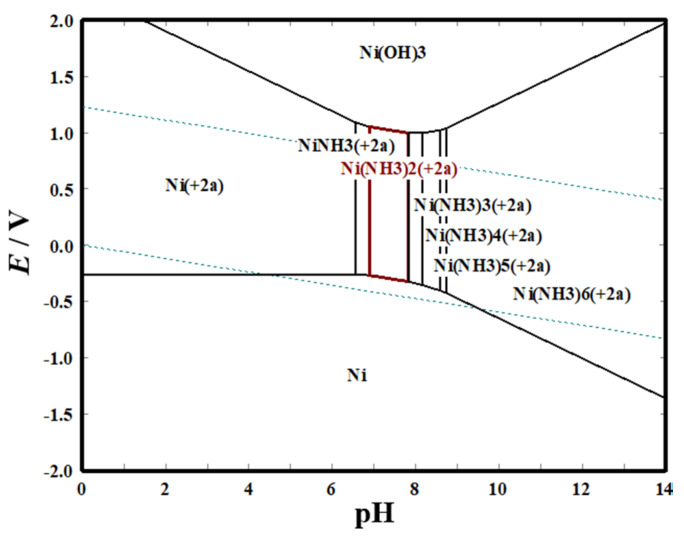
*E*-pH diagram of solution with 0.2 M NiCl_2_, 2 M NH_4_Cl, and 1 M NaCl at 25 °C.

**Figure 3:**
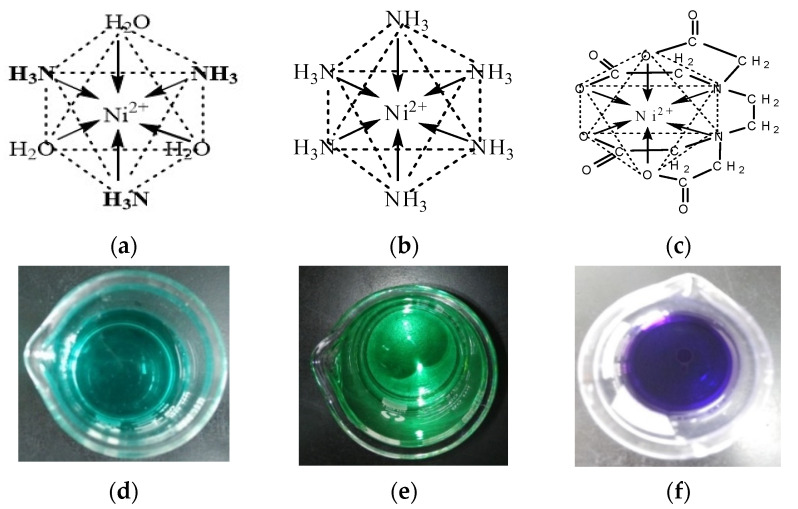
The molecular structure of different electroactive ion species for Ni electrodeposition: (**a**) Ni(NH_3_)_3_(H_2_O)_3_^2+^, (**b**) Ni(NH_3_)_6_^2+^, and (**c**) Ni chelate. The photographs of 0.2 M NiCl_2_, 2 M NH_4_Cl, and 1 M NaCl solutions with different pH values of 3 (**d**) and 7.7 (**e**), and with 0.1 M EDTA (**f**).

The equilibrium potential of metal electrodeposition changes with the electroactive ion species (i.e., molecular structure) [25], which affects the metal electrocrystallization behavior (including crystal nucleation and growth). In our previous study [16], it was found that self-supporting porous Ni film could be electrodeposited only when the NH_4_^+^ concentration reaches a certain value. According to the molecular structure characteristics of NH_4_^+^, in addition to being used as a buffer and hydrogen source, it can also be used as a complexing agent to form complexes with Ni^2+^ to change the electroactive ion species for Ni electrodeposition. As shown in the *E*-pH diagram (Figure 2), in solution containing 0.2 M NiCl_2_, 2 M NH_4_Cl, and 1 M NaCl, the molecular structure of Ni(NH_3_)*_n_*^2+^ (6 ≥ *n* ≥ 0) complex is different at various pH values. The number of NH_3_ molecules bound by Ni^2+^ in the formed Ni(NH_3_)*_n_*^2+^ (6 ≥ *n* ≥ 0) complexes gradually increases with the increase of the pH value, and the binding force of the coordination bond increases, thus the theoretical equilibrium potential shifts negatively (Figure 2, Figure 3a,b). Additionally, EDTA could chelate with Ni^2+^ to form a cyclic chelate structure which is different from Ni(NH_3_)*_n_*^2+^ complexes (Figure 3c). The six-coordinated chelate has a more stable thermodynamic structure than the Ni(NH_3_)*_n_*^2+^ complexes, so its theoretical equilibrium potential is more negative.

Based on the above theoretical analysis, different forms of electroactive ion species for Ni electrodeposition can be formed by adjusting the pH value of the basic solution (Table 1). To obtain Ni chelate, 0.1 M EDTA is added to the basic solution. As Figure 3d–f shows, the solution changes from dark green to light blue as the pH value increases from 3 to 7.7, and the solution containing 0.1 M EDTA is dark purple. The above results indicate that different electroactive ion species for Ni electrodeposition are formed under different solution conditions.

**Figure 4:**
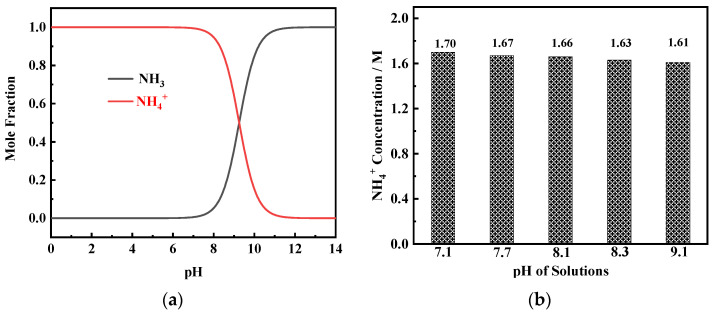
(**a**) Mole fraction–pH diagram of NH_3_-NH_4_^+^ species in solutions containing 0.2 M NiCl_2_, 2 M NH_4_Cl, and 1 M NaCl. (**b**) NH_4_^+^ concentration in 0.2 M NiCl_2_, 2 M NH_4_Cl, and 1 M NaCl solutions with different pH values.

To obtain the high pH value, a large amount of dilute NaOH solution is added to the basic solution to react with NH_4_^+^ (reaction (2)). The product NH_3_.H_2_O is decomposed into NH_3_ and H_2_O (reaction (3)). Ni^2+^ complexes react with NH_3_ to form Ni(NH_3_)*_n_*^2+^ complexes (reaction (4)) which are reduced to metal Ni (reaction (5)) during the electrodeposition process. From the mole fraction–pH diagram of NH_3_–NH_4_^+^ species (Figure 4a), it can be found that, when pH value is greater than 7, the mole fraction of NH_3_ begins to increase while the mole fraction of NH_4_^+^ begins to decrease. The results of the ion chromatography measurement show that in solutions containing 0.2 M NiCl_2_, 2 M NH_4_Cl, and 1 M NaCl, when the pH value reaches between 7.1 and 9.1, the NH_4_^+^ concentration is in the range of 1.6–1.7 M (Figure 4b). This result indicates that even in the solution with a high pH value (9.1), very little NH_3_ volatilizes, and the NH_4_^+^ concentration in the solution can reach the critical content required for porous Ni film formation [16].

(2) $$NH_{4}{}^{+}+OH^{-}=NH_{3}.H_{2}O$$

(3) $$NH_{3}.H_{2}O=NH_{3}+H_{2}O$$

(4) $$Ni^{2+}+nNH_{3}=Ni{\left(NH_{3}\right)}_{n}{}^{2+}$$

(5) $$Ni{\left(NH_{3}\right)}_{n}{}^{2+}+2e^{-}=Ni+nNH_{3}$$

**Figure 5:**
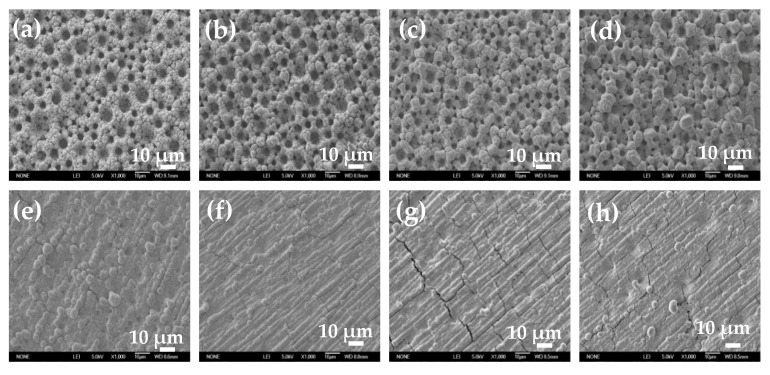
SEM images of Ni films electrodeposited in 0.2 M NiCl_2_, 2 M NH_4_Cl, and 1 M NaCl solutions with different pH values of 3 (**a**), 6.5 (**b**), 7.1 (**c**), 7.7 (**d**), 8.1 (**e**), 8.3 (**f**), and 9.1 (**g**), and with 0.1 M EDTA (**h**). Magnification: 1000×.

**Figure 6:**
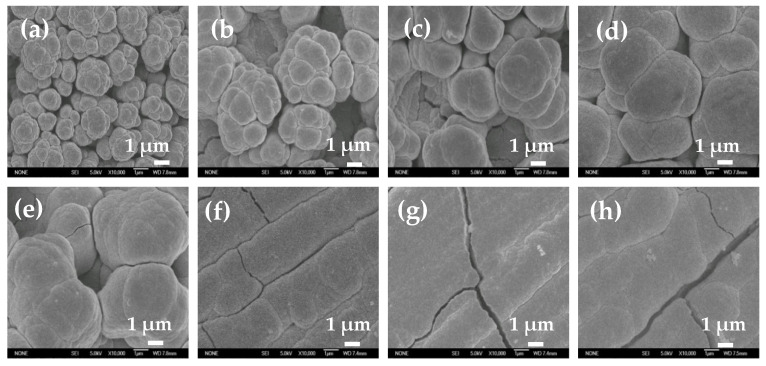
SEM images of Ni films electrodeposited in 0.2 M NiCl_2_, 2 M NH_4_Cl, and 1 M NaCl solutions with different pH values of 3 (**a**), 6.5 (**b**), 7.1 (**c**), 7.7 (**d**), 8.1 (**e**), 8.3 (**f**), and 9.1 (**g**), and with 0.1 M EDTA (**h**). Magnification: 10,000×.

To study the influence of electroactive ion species on the surface structure of electrodeposited Ni film, Ni films were electrodeposited in solutions with different pH values or containing 0.1 M EDTA at −1 A cm^−2^ for 30 s. SEM images of electrodeposited Ni films at two different magnifications are shown in Figure 5 and Figure 6. When the pH value is less than 7.7, self-supporting Ni films with uniform porous structure can be electrodeposited (Figure 5a–d). With the increase of the pH value, the diameter of the pore decreases, and the pore wall becomes thicker. When the pH value reaches 8.1, the porous structure degenerates and disappears, and a rough Ni film composed of agglomerated particles appears on the electrode surface (Figure 5e). When the pH value further increases (pH = 8.3), the number of the agglomerated particle decreases and the size of the agglomerated particle becomes smaller (Figure 5f). When the pH value reaches 9.1, the agglomerated particles disappear, and a flat and compact Ni film is formed. It is worth noting that Ni film obtained by reducing the chelate in basic solution with 0.1 M EDTA is flatter and more compact, without obvious agglomerated particles on the surface (Figure 5h).

Figure 6 is the magnified image of the corresponding SEM image in Figure 5. When the pH value is 3 or 6.5, smaller size Ni particles with dispersed arrangement are obtained. When the pH value is greater than 7.1, Ni particles are tightly agglomerated together. As the pH value further increases, Ni particles gradually become larger. When the pH value is higher than 8.3, the surface of the Ni film is flat and compact, without obvious particle boundaries. The Ni film obtained by reducing the chelate is flatter and more compact, without obvious particle boundaries. At the same time, it should be noted that there are cracks on the surface of flat and compact Ni films (Figure 5g,h, Figure 6f,h).

**Figure 7:**
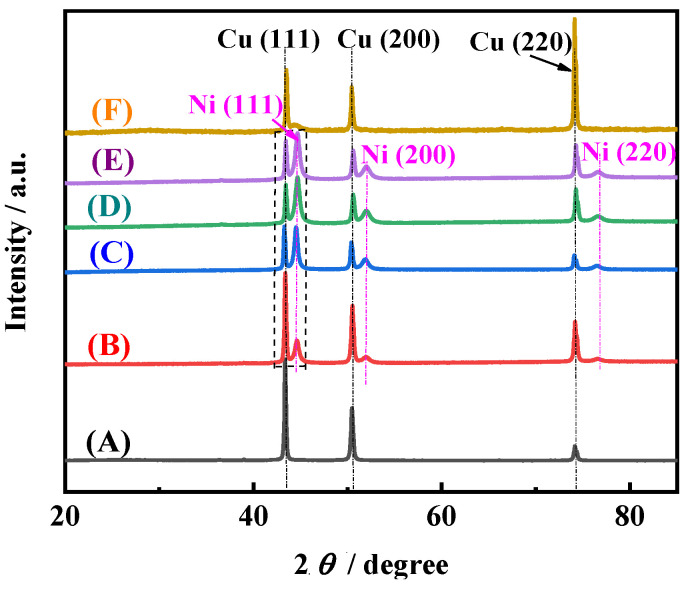
The XRD patterns of different films: Cu substrate (A) and Ni films electrodeposited in 0.2 M NiCl_2_, 2 M NH_4_Cl, and 1 M NaCl solutions with different pH values of 3 (B), 7.1 (C), 8.1 (D), and 9.1 (E), and with 0.1 M EDTA (F).

Figure 7 shows the typical XRD patterns of electrodeposited Ni films. It can be seen that for the Ni films electrodeposited in EDTA-free solutions with different pH values, except for the three peaks Cu(111), Cu(200), and Cu(220) of the Cu substrate [26], three peaks Ni(111), Ni(200), and Ni(220) of electrodeposited Ni films appear [2], which is consistent with the previous results of the electrodeposition Ni [2]. The crystal plane Ni(111) is the preferred orientation, and the intensity of three characteristic peaks for Ni films increases with the increase of the pH value. It is worth noting that, for Ni film electrodeposited in solution with 0.1 M EDTA, only a broad and less sharp peak appears in the range of 44°–45°. It shows that amorphous Ni is obtained [7], which may be because EDTA changes the electrocrytallization mechanism. The above results indicate that pH value and EDTA not only affect the surface morphology but also the crystal structure of the electrodeposited Ni film.

**Figure 8:**
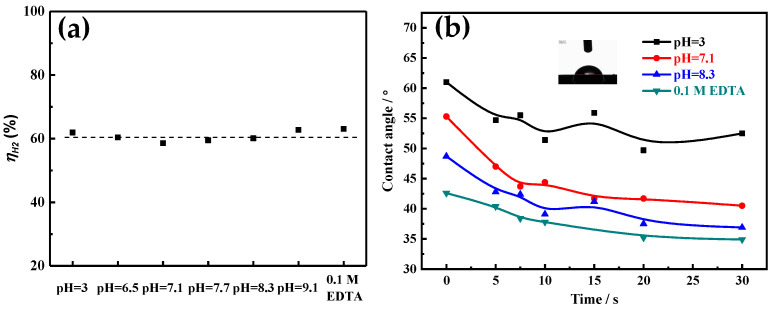
(**a**) The current efficiencies of HER for different Ni electrodeposition solutions. (**b**) Time-dependent interface contact angle (*Φ*) evolution of different solutions during the Ni electrodeposition process.

The current efficiency of hydrogen evolution (i.e., bubble evolution amount) can affect the electrocrystallization behaviors (especially crystal growth) by changing the electrodeposition dynamics condition near the electrode surface, which can determine the morphology and size of the electrodeposited metal particle [20]. Additionally, the porous structure of the electrodeposited Ni film is related to the behavior (such as bubble nucleation, coalescence, growth and detachment) of the bubble on the substrate surface [2,19]. Therefore, during the Ni electrodeposition process, the current efficiency of hydrogen evolution in different solutions is calculated based on the mass of electrodeposited Ni. As shown in Figure 8a, it can be found that in acid region (3 ≤ pH ≤7.1), the current efficiency of hydrogen evolution slightly decreases with the increase of the pH value. Yet in the alkaline region (7.1 ≤ pH ≤ 9.1), the current efficiency of hydrogen evolution slightly increases with the increase of the pH value. For the solution with 0.1 M EDTA, the maximum current efficiency of hydrogen evolution is obtained. However, for different solutions, the current efficiency of hydrogen evolution is about 60%. That is, the form of electroactive ion species used for Ni electrodeposition hardly affects the current efficiency of hydrogen evolution.

Furthermore, bubble size and bubble residence time on the substrate surface ultimately rely on the break-off diameter (*d*) which is expressed by Formula (6) [2,19,27]:

(6) $$d=0.02\phi {\left(\frac{\gamma }{g(\rho_{l}-\rho_{g})}\right)}^{0.5}{\left(1+0.2\frac{i}{Am^{-2}}\right)}^{-0.45}$$

where *Φ* is the contact angle (°), *g* is the gravity acceleration (9.8 m s^−1^), *γ* is the surface tension (mN m^−1^), *i* is the current density (A cm^−2^), *ρ*_l_ and *ρ*_g_ are solution density (kg dm^−3^) and gas density (kg dm^−3^), respectively. 

It can be seen from Formula (6) that at a high current density, the bubble break-off diameter is mainly determined by the interface wettability characteristic, such as contact angle (*Φ*) and surface tension (*γ*). To accurately calculate the real-time break-off diameter of the adsorption bubble during the electrodeposition process, the real-time contact angle is measured, which is related to the surface condition of the electrode. As Figure 8b shows, contact angles of all electrodeposited Ni films decrease significantly in the first 10 s, and then tend to level off. It also can be found that the contact angle decreases with the increase of the pH value, and the solution with 0.1 M EDTA possesses the smallest contact angle. The surface tension of different solutions is also measured and shown in Table 2. The surface tension values are all around 76.5 mN m^−1^, with little difference. According to Formula (6), the bubble break-off diameter is calculated based on the measurement parameter values in Figure 8b and Table 2. As Table 2 shows, the bubble break-off diameter decreases with the increase of the pH value. For example, it decreases from 154.34 μm at pH = 3 to 117.29 μm at pH = 8.3. In solution with 0.1 M EDTA, the minimum bubble break-off diameter is obtained, which is about 109.91 μm. As the insulating hydrogen bubbles adsorbed on the film surface are used as a porous structure template, the pore diameter depends on the size of the bubble. So, the pore diameter of Ni film decreases with the increase of the pH value (Figure 5).

**Figure 9:**
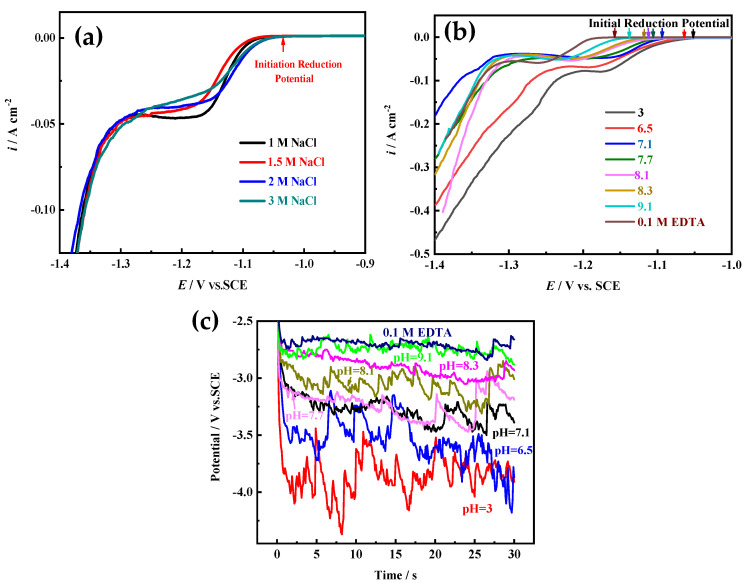
(**a**) The polarization curves of Ni electrodeposition in solutions (pH = 3) with different NaCl concentrations at 10 mV s^−1^. (**b**) The polarization curves of Ni electrodeposition in solutions with different pH values or components at 10 mV s^−1^. (**c**) The chronopotentiometry (CP) curves of Ni electrodeposition in solutions with different pH values or components at −1 A cm^−2^.

On the other hand, electrocrystallization behavior (including crystal nucleation and growth) is also an important factor for determining the structure of electrodeposited film, which greatly relies on the electroactive ion species for metal electrodeposition and the electrode reaction kinetic conditions [15,16]. According to the above research, different Ni electroactive ion species are formed in solutions with different pH values or components (Figure 2, Figure 3 and Figure 4). To reveal the microstructure evolution mechanism of Ni films electrodeposited in solutions with different pH values or 0.1 M EDTA, the polarization curves of Ni electrodeposition in different solutions are measured at 10 mV s^−1^. Moreover, the influence of NaCl concentration on the polarization curve of Ni electrodeposition is also investigated, because by using NaOH for pH adjustment, a large amount of NaCl would be produced to obtain a higher pH value (pH ≥ 7.1). As Figure 9a shows, the location of the initial reduction potential and peak potential of Ni electrodeposition has hardly changed in solutions containing 0.2 M NiCl_2_ and 2 M NH_4_Cl and different NaCl concentrations, indicating that NaCl concentration has hardly any effect on Ni electrocrystallization behavior. From the results of Figure 9b, it can be found that the initial reduction potentials and peak potentials of Ni electrodeposition shift toward a negative direction with the increase of the pH value, and in solution with 0.1 M EDTA, the most negative initial reduction potential for Ni electrodeposition is used. The results indicate that with the increase of the pH value, the Ni electroactive ion species change from Ni^2+^ to Ni(NH_3_)*_n_*^2+^ (6 ≥ n ≥ 0) complexes, and as the number of NH_3_ molecules (i.e., n) bound in the Ni(NH_3_)*_n_*^2+^ complexes increases, the reduction equilibrium potential gradually shifts more negative. Ni electrodeposited in solution containing 0.1 M EDTA needs the most negative equilibrium potential. 

According to the metal electrocrystallization nucleation theory, the relationships of nucleation formation energy *A* (J mol^−1^), nucleation probability *W,* and overpotential *η* (V) are expressed as [28]:

(7) $$A=\frac{16\pi \sigma^{2}V^{2}}{3z^{2}F^{2}\eta_{}^{2}}$$

(8) $$W=B\exp(-b/\eta^{2})$$

(9) $$zF\eta_{}=2\sigma V/h_{i}$$

where *σ* is interfacial free energy (J m^−2^), *V* is molar volume (m^3^ mol^−1^), *z* is the number of transferred electrons, *F* is the Faraday constant (96485 C mol^−1^), *B* and *b* are constants, *h*_i_ is critical nucleation radius (m). According to Equations (7)–(9), with the decrease of overpotential (i.e., the increase of the pH or adding EDTA), the nucleation formation energy and critical nucleation radius increase, and nucleation probability decreases. Therefore, the growth probability and size of electrodeposited Ni crystal increases. This conclusion is also confirmed by the results of SEM and XRD measurements in Figure 5, Figure 6 and Figure 7. That is, in EDTA-free solutions with low pH value, due to the high overpotential of Ni electroactive ion species, the particles with small size are easy to generate, leading to the formation of a rough porous structure which exhibits a large surface area and good wettability. In solutions with high pH value or 0.1 M EDTA, the Ni(NH_3_)*_n_*^2+^ complexes or Ni-EDTA chelate are formed, which can inhibit the nucleation of Ni electrocrystallization, and enhance the leveling effect of isotropic growth of the electrodeposited film. Consequently, the electrodeposited Ni film grows uniformly without forming protrusion particles.

**Figure 10:**
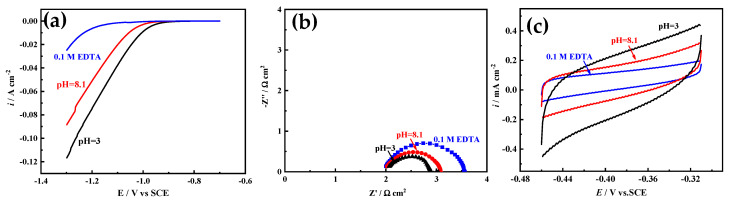
(**a**) The polarization curves at 10 mV s^−1^ and (**b**) the Nyquist plots at 250 mV overpotential of Ni films electrodeposited in different solutions in 1 M NaOH for HER. (**c**) The cyclic voltammetry curves of Ni films with a potential range from −0.31 to −0.46 V at 10 mV s^−1^ in 1 M NaOH.

Ni film is usually used as a catalytic electrode or current collector for hydrogen evolution reaction (HER) [3]. The electrocatalytic activity area is an important factor affecting the HER activity. Herein, Ni films electrodeposited in acid solution (pH = 3), alkaline solution (pH = 8.1), and solution with 0.1 M EDTA are selected to study the HER electrocatalytic activity, and the electrocatalytic activity area is simply evaluated based on EIS and CV measurements [29,30]. As Figure 10a shows, with the increase of pH value, the HER electrocatalytic activity of Ni film decreases, and for Ni film electrodeposited in solution with 0.1 M EDTA, the worst HER electrocatalytic activity appears. It is well known that the electrocatalytic activity area of the Ni film is inversely proportional to the semicircle diameter of the Nyquist plot [29] (that is, proportional to the electrochemical surface roughness), and proportional to the area covered by the CV curve [30] (that is, proportional to the electric double layer capacitance). As shown in Figure 10b,c, with the increase of pH value, the semicircle diameter increases, and the area covered by the CV curve decreases, indicating that the catalytic activity area of the film decreases. In solution with 0.1 M EDTA, the semicircle diameter is the largest and the area covered by the CV curve is the smallest, exhibiting that the catalytic activity area of the film is the smallest, which is consistent with the surface structure of Ni films (Figure 5 and Figure 6). The BET measurement also shows that the particle of the Ni film electrodeposited in acid solution (pH = 3) possesses the largest special surface area (about 125 m^2^ g^−1^), which are much larger than those of Ni films electrodeposited in alkaline solution (pH = 8.1) (about 91 m^2^ g^−1^) and solution with 0.1 M EDTA (about 47 m^2^ g^−1^).

**Figure 11:**
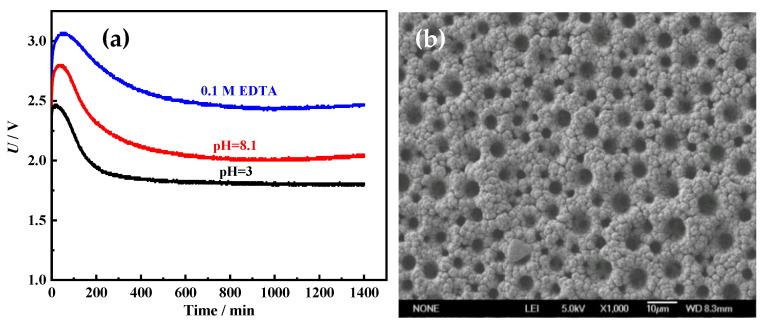

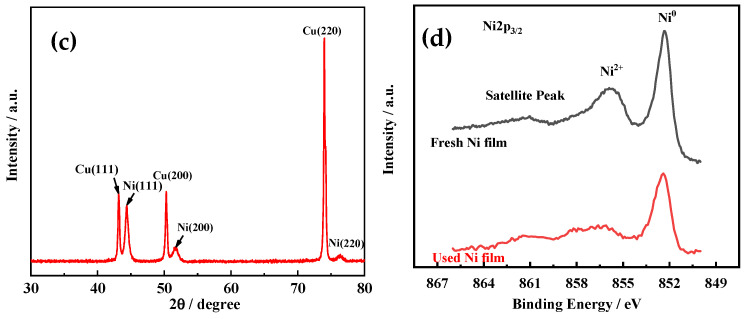
(**a**) The cell voltage (U)–time (t) curves of the Ni films at 100 mA cm^−2^. The SEM image (**b**) and XRD pattern (**c**) of the Ni film electrodeposited in acid solution (pH = 3) after the long-term electrolysis. (**d**) XPS spectra for the fresh Ni film electrodeposited in acid solution (pH = 3) and the Ni film after the long-term electrolysis.

To investigate the stability of the Ni films for HER, the long-term electrolysis was performed in 1 M NaOH at 100 mA cm^−2^ for 1400 min. The cell voltage–time (U–t) curves were recorded and are shown in Figure 11a. Cell voltages of all Ni films increase firstly, and then drop. After about 300 min, cell voltages are almost unchanged. It means that all porous metal films possess good long-term stability. In addition, it is also found that the cell voltages of porous Ni film electrodeposited in acid solution (pH = 3) (about 1.85 V) are much lower than those of Ni films electrodeposited alkaline solution (pH = 8.1) (about 2.15 V) and solution with 0.1 M EDTA (about 2.55 V). It proves once again that the Ni film electrodeposited in acid solution (pH = 3) possesses the best HER performance. From the SEM image (Figure 11b) and XRD pattern (Figure 11c) of the Ni film electrodeposited in acid solution (pH = 3) after the long-term electrolysis, it can be found that the morphology and crystal structure are almost unchanged. Through the comparison of the XPS spectra for the fresh Ni film electrodeposited in acid solution (pH = 3) and the Ni film after the long-term electrolysis, it also can be found that the surface chemical compositions and states of the Ni film before and after use are almost the same (Figure 11d). The Ni film surface is mainly composed of metallic Ni and a small amount of Ni oxide (Ni^2+^) [29]. The above results indicate that the Ni film exhibits excellent long-term catalytic stability for HER.

## 4. Conclusions

The structure evolution mechanism of electrodeposited Ni films is studied based on the adjustment of the pH value and EDTA additive on the hydrogen evolution side reaction and metal electrocrystallization behavior. It was found that in solutions with low pH value, since Ni^2+^ with a high reduction overpotential is the electroactive ion, it is easy to generate small-sized particles, thereby forming a rough porous structure. Moreover, due to the large contact angle of the solution on the substrate, hydrogen bubbles with a large break-off diameter adsorb on the substrate being used as a template for the porous structure. As a result, porous Ni films with large pore diameters are formed. With the increase of the pH value, the number of NH_3_ bound by Ni^2+^ in the formed Ni(NH_3_)*_n_*^2+^ (6 ≥ n ≥ 0) complexes gradually increases. Additionally, the binding force of the coordination bond increases, leading to a negative shift of the equilibrium potential (i.e., initial reduction potential) for Ni electrodeposition. Furthermore, EDTA combines Ni^2+^ to form a more stable cyclic chelate structure, and the equilibrium potential of Ni electrodeposition is more negative. However, at −1 A cm^−2^, as the pH value increases, the electrode potential for Ni reduction shifts positive (i.e., the overpotential decreases). The reduction of the chelate to metal Ni requires a lower overpotential. In addition, due to the improvement of wettability, hydrogen bubbles with small break-off diameters rapidly detach the film and produce strong stirring to improve the electrode reaction kinetic conditions. Ni(NH_3_)*_n_*^2+^ complexes and Ni–EDTA chelate can inhibit the nucleation of Ni electrocrystallization, and have the leveling effect of isotropic growth of the electrodeposited Ni film (i.e., uniform growth without forming protruding particles). With the increase of the structural stability of the Ni electroactive ion species, the ability of inhibiting nucleation and leveling of the electrodeposited film gradually increases. Finally, the surface morphology of the Ni film evolves from a porous structure composed of small particles to a flat and compact film with larger particles.

## Figures and Tables

**Table 1:** The theoretical (based on the *E*-pH diagram in Figure 2 and experimental pH values of solution with 0.2 M NiCl_2_, 2 M NH_4_Cl, and 1 M NaCl for the formation of different Ni(NH_3_)*_n_*^2+^ complexes.

Species	Ni^2+^	Ni(NH_3_)^2+^	Ni(NH_3_)_2_^2+^	Ni(NH_3_)_3_^2+^	Ni(NH_3_)_4_^2+^	Ni(NH_3_)_5_^2+^	Ni(NH_3_)_6_^2+^
Theoretical pH Range	0–6.2	6.2–6.6	6.6–7.5	7.5–7.8	7.8–8.3	8.3–8.4	8.4–14
Experimental pH	3	6.5	7.1	7.7	8.1	8.3	9.1

**Table 2:** The density (*ρ*), surface tension (*γ*), and break-off diameter (*d*) of Ni electrodeposition solutions with different pH values and components.

Solution	pH = 3	pH = 7.1	pH = 8.3	0.1 M EDTA
*ρ* (kg dm^−3^)	1.07922	1.07901	1.08463	1.0917
*r* (mN m^−1^)	76.31	76.88	76.27	76.76
*d* (μm)	154.34	125.41	117.29	109.91
